# Do we measure gray matter activation with functional diffusion tensor imaging?

**DOI:** 10.3389/fnins.2014.00126

**Published:** 2014-05-27

**Authors:** René C. W. Mandl, Hugo G. Schnack, Marcel P. Zwiers, René S. Kahn, Hilleke E. Hulshoff Pol

**Affiliations:** ^1^Department of Psychiatry, Brain Center Rudolf Magnus, University Medical Center UtrechtUtrecht, Netherlands; ^2^Centre for Cognitive Neuroimaging, Donders Institute for Brain, Cognition, and Behaviour, Radboud University NijmegenNijmegen, Netherlands

**Keywords:** white matter activation, BOLD signal, partial volume effects, signal leaking, fiber tracts

In this comment by Autio and Roberts (2014) on our second fDTI article “Functional Diffusion Tensor Imaging at 3 Tesla” (Mandl et al., 2013) the authors suggest that BOLD signal originating from gray matter could in part explain the reported task-related FA changes in white matter. The rationale is that the relative contribution from activated gray matter to the measured signal increases in voxels containing both gray and white matter. Because the ADC value for gray matter is between the parallel and perpendicular ADC for white matter, this increased contribution effectively could lead to an increase in the measured parallel ADC and a decrease in the measured perpendicular ADC and hence an increase in FA. Indeed, contamination by signal “leaking” from gray matter into white matter has been one of our major concerns in both our fDTI articles, together with the effects of motion.

However, we think that the proposed mechanism by Autio and Roberts to the reported task-related FA changes does not contribute to our finding. One, the use of the non-parametric sign test in our first fDTI paper (Mandl et al., 2008) prevents that only a few voxels (e.g., the end points of the tract touching active gray matter) can result in activation of a complete tract. Two, the global shift of the histograms presented in Figure 5 Mandl et al. (2008) shows that a large part of the white matter voxels in the active tracts contribute to the measured task-related FA change. Of course this in itself does not rule out the proposed mechanism because it could be that the active tracts are (for a large part) surrounded by active gray matter voxels. This may for instance be the case for the optic radiations. These tracts are relatively short and are for a large part adjacent to (possible active) gray matter voxels. However, this certainly is not the case for the active thalamo-cortical tracts as can be seen in the supplementary movie (Mandl et al., 2008, Movie S1). This movie shows the combined fDTI and BOLD fMRI results for the tactile experiment in a single subject (subject nr 5). It can be readily seen that the hypothesized partial voluming with possible active gray matter could only occur at the endpoints of the fiber bundle. Furthermore, three, in the second fDTI paper (Mandl et al., 2013) we introduce a time lag between the stimulus and the start of the acquisition of a fDTI volume to make the measurement less sensitive to relatively fast varying signal changes (e.g., BOLD related signal changes). Still, similar effects were reported for the tactile experiment.

Taken together we conclude that although the hypothesized mechanism by Autio and Roberts is intriguing and more experiments are needed to obtain better insight in the underlying mechanisms it cannot explain our measured task-related changes in FA in functional Diffusion Tensor Imaging.

## Conflict of interest statement

The authors declare that the research was conducted in the absence of any commercial or financial relationships that could be construed as a potential conflict of interest.
